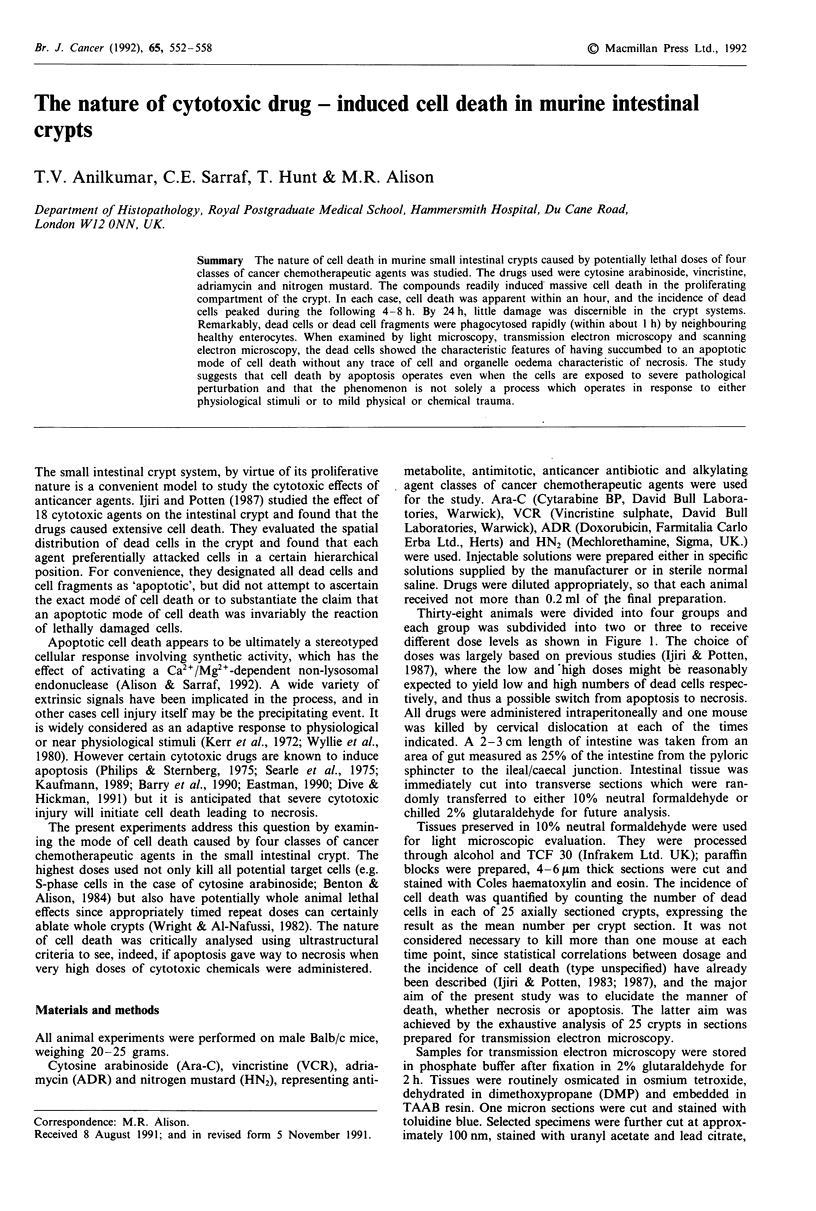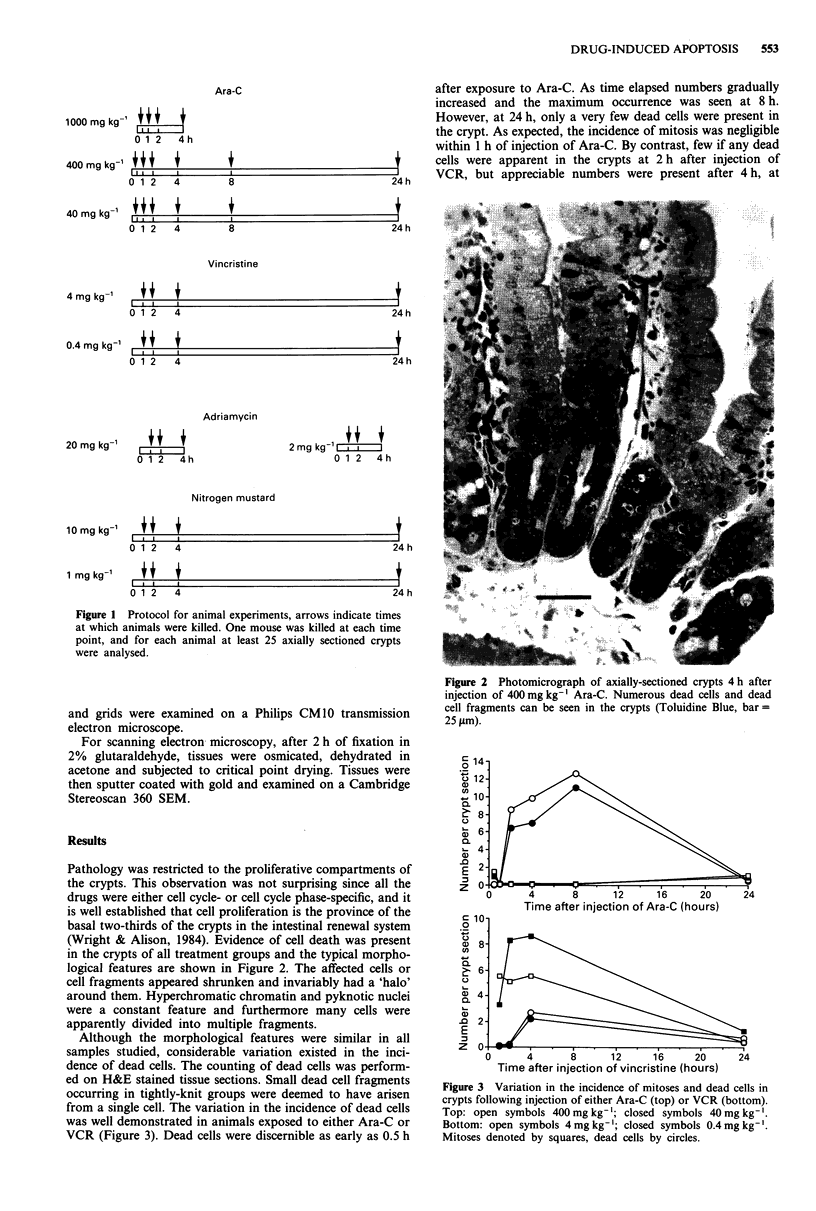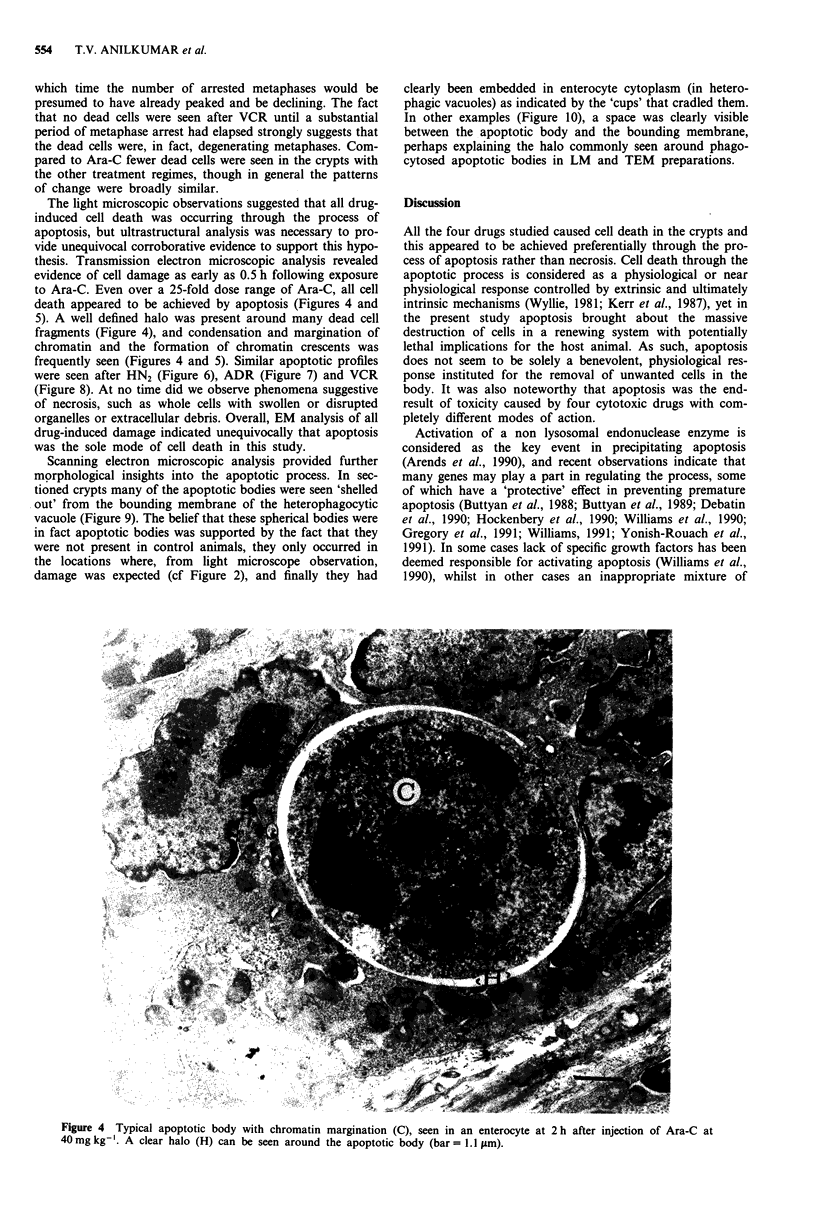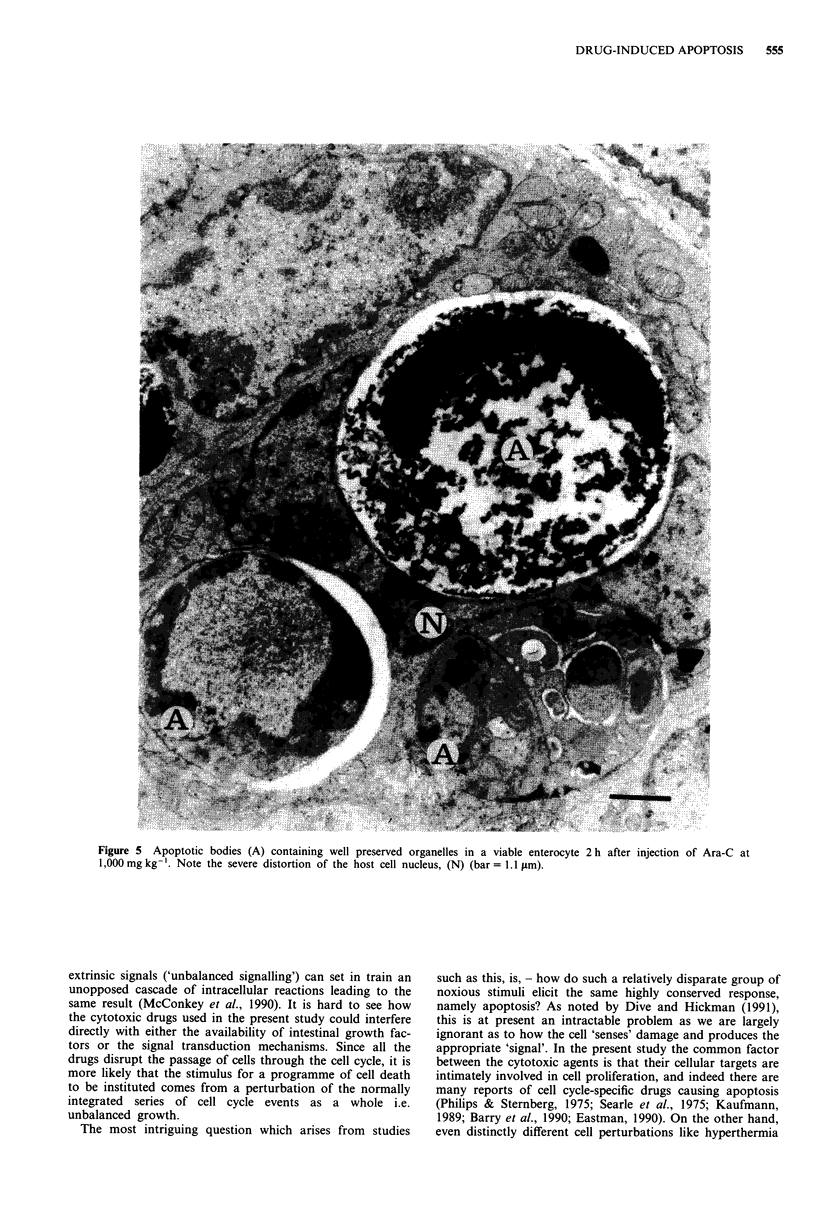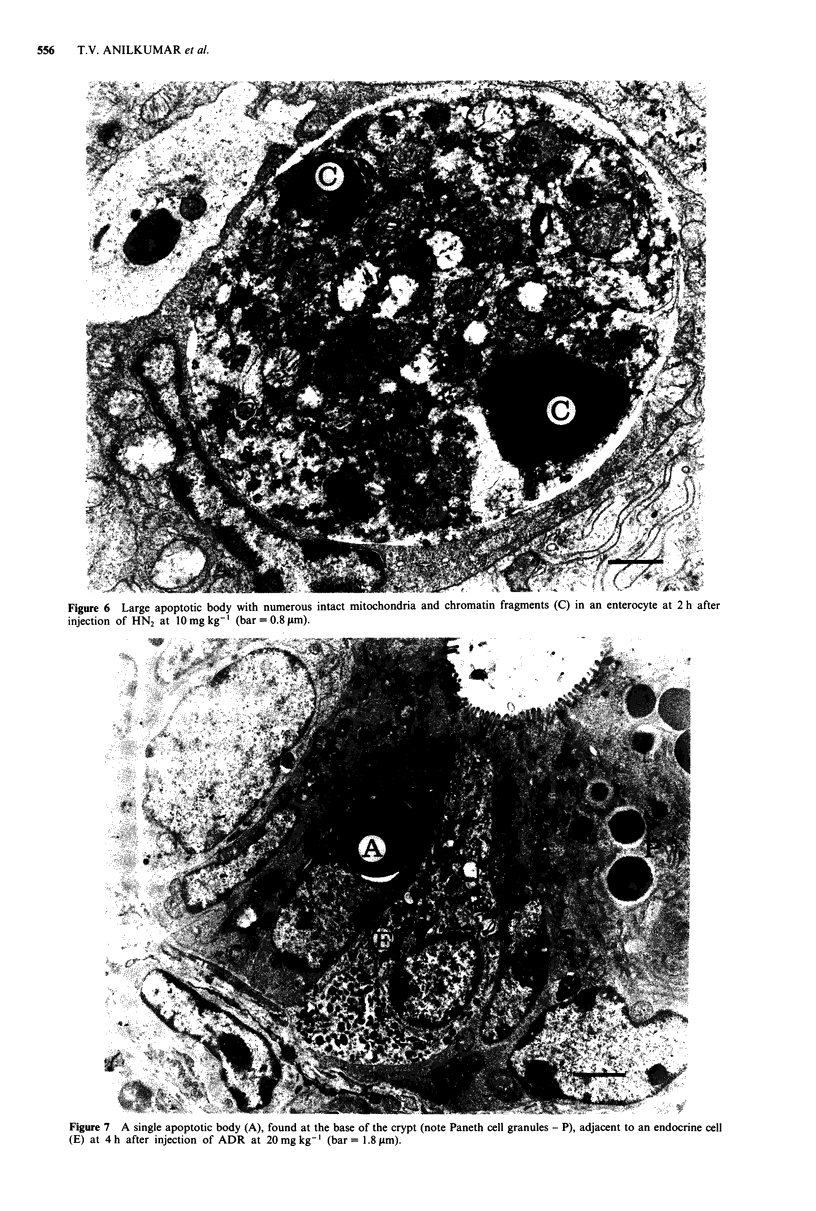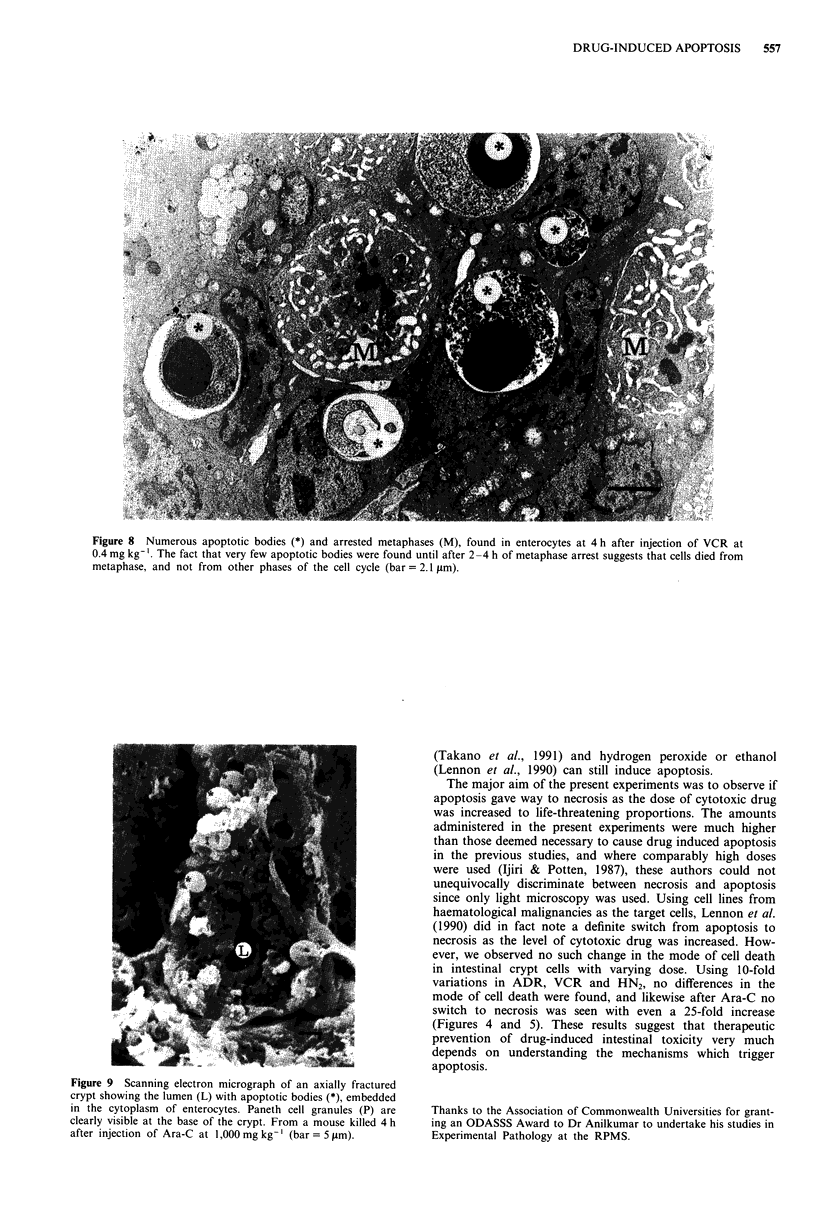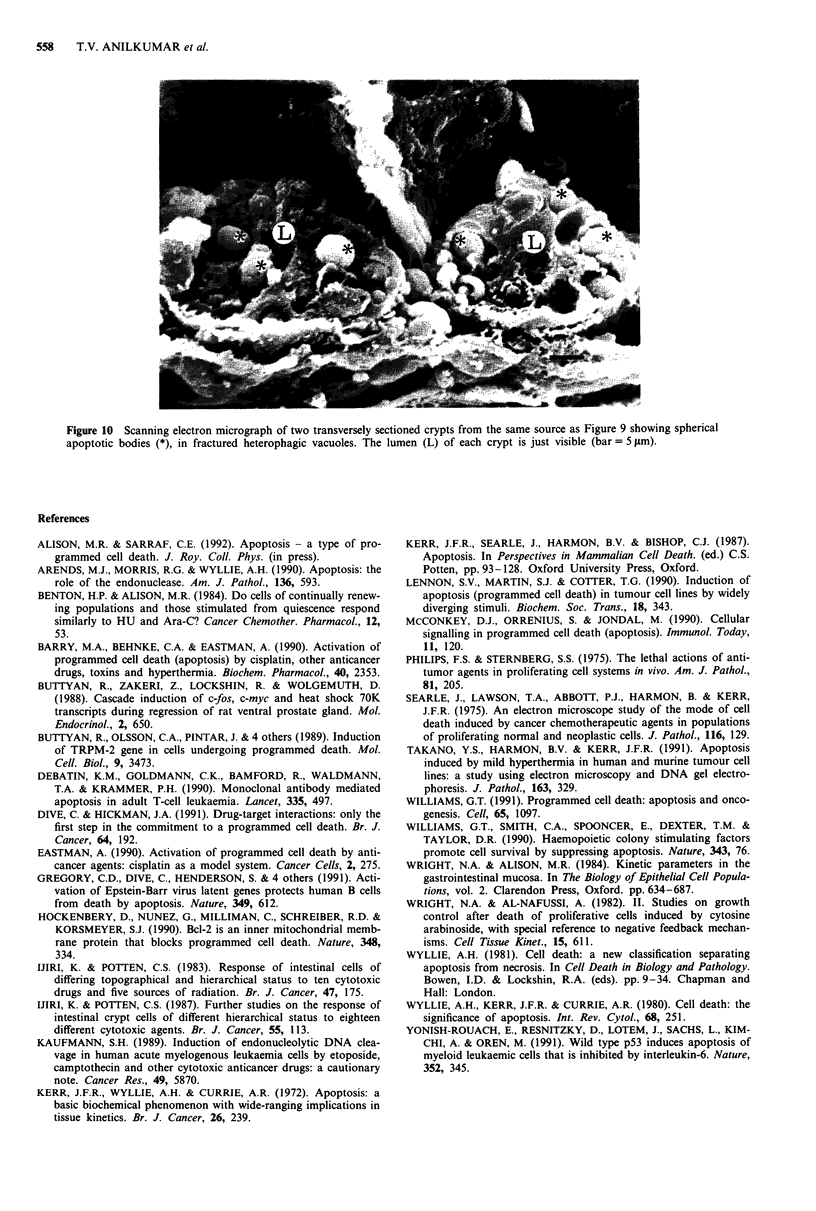# The nature of cytotoxic drug-induced cell death in murine intestinal crypts.

**DOI:** 10.1038/bjc.1992.113

**Published:** 1992-04

**Authors:** T. V. Anilkumar, C. E. Sarraf, T. Hunt, M. R. Alison

**Affiliations:** Department of Histopathology, Royal Postgraduate Medical School, Hammersmith Hospital, London, UK.

## Abstract

**Images:**


Br. J. Cancer (1992), 65, 552–558 © Macmillan Press Ltd., 1992

### The nature of cytotoxic drug – induced cell death in murine intestinal crypts

T.V. Anilkumar, C.E. Sarraf, T. Hunt & M.R. Alison

*Department of Histopathology, Royal Postgraduate Medical School, Hammersmith Hospital, Du Cane Road, London W12 0NN, UK.*

**Summary** The nature of cell death in murine small intestinal crypts caused by potentially lethal doses of four classes of cancer chemotherapeutic agents was studied. The drugs used were cytosine arabinoside, vincristine, adriamycin and nitrogen mustard. The compounds readily induced massive cell death in the proliferating compartment of the crypt. In each case, cell death was apparent within an hour, and the incidence of dead cells peaked during the following 4–8 h. By 24 h, little damage was discernible in the crypt systems. Remarkably, dead cells or dead cell fragments were phagocytosed rapidly (within about 1 h) by neighbouring healthy enterocytes. When examined by light microscopy, transmission electron microscopy and scanning electron microscopy, the dead cells showed the characteristic features of having succumbed to an apoptotic mode of cell death without any trace of cell and organelle oedema characteristic of necrosis. The study suggests that cell death by apoptosis operates even when the cells are exposed to severe pathological perturbation and that the phenomenon is not solely a process which operates in response to either physiological stimuli or to mild physical or chemical trauma.

The small intestinal crypt system, by virtue of its proliferative nature is a convenient model to study the cytotoxic effects of anticancer agents. Ijiri and Potten (1987) studied the effect of 18 cytotoxic agents on the intestinal crypt and found that the drugs caused extensive cell death. They evaluated the spatial distribution of dead cells in the crypt and found that each agent preferentially attacked cells in a certain hierarchical position. For convenience, they designated all dead cells and cell fragments as 'apoptotic', but did not attempt to ascertain the exact mode of cell death or to substantiate the claim that an apoptotic mode of cell death was invariably the reaction of lethally damaged cells.

Apoptotic cell death appears to be ultimately a stereotyped cellular response involving synthetic activity, which has the effect of activating a $Ca^{2+}/Mg^{2+}$-dependent non-lysosomal endonuclease (Alison & Sarraf, 1992). A wide variety of extrinsic signals have been implicated in the process, and in other cases cell injury itself may be the precipitating event. It is widely considered as an adaptive response to physiological or near physiological stimuli (Kerr *et al.*, 1972; Wyllie *et al.*, 1980). However certain cytotoxic drugs are known to induce apoptosis (Philips & Sternberg, 1975; Searle *et al.*, 1975; Kaufmann, 1989; Barry *et al.*, 1990; Eastman, 1990; Dive & Hickman, 1991) but it is anticipated that severe cytotoxic injury will initiate cell death leading to necrosis.

The present experiments address this question by examining the mode of cell death caused by four classes of cancer chemotherapeutic agents in the small intestinal crypt. The highest doses used not only kill all potential target cells (e.g. S-phase cells in the case of cytosine arabinoside; Benton & Alison, 1984) but also have potentially whole animal lethal effects since appropriately timed repeat doses can certainly ablate whole crypts (Wright & Al-Nafussi, 1982). The nature of cell death was critically analysed using ultrastructural criteria to see, indeed, if apoptosis gave way to necrosis when very high doses of cytotoxic chemicals were administered.

### Materials and methods

All animal experiments were performed on male Balb/c mice, weighing 20–25 grams.

Cytosine arabinoside (Ara-C), vincristine (VCR), adriamycin (ADR) and nitrogen mustard ($HN_2$), representing antimetabolite, antimitotic, anticancer antibiotic and alkylating agent classes of cancer chemotherapeutic agents were used for the study. Ara-C (Cytarabine BP, David Bull Laboratories, Warwick), VCR (Vincristine sulphate, David Bull Laboratories, Warwick), ADR (Doxorubicin, Farmitalia Carlo Erba Ltd., Herts) and $HN_2$ (Mechlorethamine, Sigma, UK.) were used. Injectable solutions were prepared either in specific solutions supplied by the manufacturer or in sterile normal saline. Drugs were diluted appropriately, so that each animal received not more than 0.2 ml of the final preparation.

Thirty-eight animals were divided into four groups and each group was subdivided into two or three to receive different dose levels as shown in Figure 1. The choice of doses was largely based on previous studies (Ijiri & Potten, 1987), where the low and high doses might be reasonably expected to yield low and high numbers of dead cells respectively, and thus a possible switch from apoptosis to necrosis. All drugs were administered intraperitoneally and one mouse was killed by cervical dislocation at each of the times indicated. A 2–3 cm length of intestine was taken from an area of gut measured as 25% of the intestine from the pyloric sphincter to the ileal/caecal junction. Intestinal tissue was immediately cut into transverse sections which were randomly transferred to either 10% neutral formaldehyde or chilled 2% glutaraldehyde for future analysis.

Tissues preserved in 10% neutral formaldehyde were used for light microscopic evaluation. They were processed through alcohol and TCF 30 (Infrakem Ltd. UK); paraffin blocks were prepared, 4–6 µm thick sections were cut and stained with Coles haematoxylin and eosin. The incidence of cell death was quantified by counting the number of dead cells in each of 25 axially sectioned crypts, expressing the result as the mean number per crypt section. It was not considered necessary to kill more than one mouse at each time point, since statistical correlations between dosage and the incidence of cell death (type unspecified) have already been described (Ijiri & Potten, 1983; 1987), and the major aim of the present study was to elucidate the manner of death, whether necrosis or apoptosis. The latter aim was achieved by the exhaustive analysis of 25 crypts in sections prepared for transmission electron microscopy.

Samples for transmission electron microscopy were stored in phosphate buffer after fixation in 2% glutaraldehyde for 2 h. Tissues were routinely osmicated in osmium tetroxide, dehydrated in dimethoxypropane (DMP) and embedded in TAAB resin. One micron sections were cut and stained with toluidine blue. Selected specimens were further cut at approximately 100 nm, stained with uranyl acetate and lead citrate,

Correspondence: M.R. Alison.
Received 8 August 1991; and in revised form 5 November 1991.

**Figure 1** Protocol for animal experiments, arrows indicate times at which animals were killed. One mouse was killed at each time point, and for each animal at least 25 axially sectioned crypts were analysed.

and grids were examined on a Philips CM10 transmission electron microscope.

For scanning electron microscopy, after 2 h of fixation in 2% glutaraldehyde, tissues were osmicated, dehydrated in acetone and subjected to critical point drying. Tissues were then sputter coated with gold and examined on a Cambridge Stereoscan 360 SEM.

### Results

Pathology was restricted to the proliferative compartments of the crypts. This observation was not surprising since all the drugs were either cell cycle- or cell cycle phase-specific, and it is well established that cell proliferation is the province of the basal two-thirds of the crypts in the intestinal renewal system (Wright & Alison, 1984). Evidence of cell death was present in the crypts of all treatment groups and the typical morphological features are shown in Figure 2. The affected cells or cell fragments appeared shrunken and invariably had a 'halo' around them. Hyperchromatic chromatin and pyknotic nuclei were a constant feature and furthermore many cells were apparently divided into multiple fragments.

Although the morphological features were similar in all samples studied, considerable variation existed in the incidence of dead cells. The counting of dead cells was performed on H&E stained tissue sections. Small dead cell fragments occurring in tightly-knit groups were deemed to have arisen from a single cell. The variation in the incidence of dead cells was well demonstrated in animals exposed to either Ara-C or VCR (Figure 3). Dead cells were discernible as early as 0.5 h after exposure to Ara-C. As time elapsed numbers gradually increased and the maximum occurrence was seen at 8 h. However, at 24 h, only a very few dead cells were present in the crypt. As expected, the incidence of mitosis was negligible within 1 h of injection of Ara-C. By contrast, few if any dead cells were apparent in the crypts at 2 h after injection of VCR, but appreciable numbers were present after 4 h, at

**Figure 2** Photomicrograph of axially-sectioned crypts 4 h after injection of 400 mg kg$^{-1}$ Ara-C. Numerous dead cells and dead cell fragments can be seen in the crypts (Toluidine Blue, bar = 25 $\mu$m).

**Figure 3** Variation in the incidence of mitoses and dead cells in crypts following injection of either Ara-C (top) or VCR (bottom). Top: open symbols 400 mg kg$^{-1}$; closed symbols 40 mg kg$^{-1}$. Bottom: open symbols 4 mg kg$^{-1}$; closed symbols 0.4 mg kg$^{-1}$. Mitoses denoted by squares, dead cells by circles.

which time the number of arrested metaphases would be presumed to have already peaked and be declining. The fact that no dead cells were seen after VCR until a substantial period of metaphase arrest had elapsed strongly suggests that the dead cells were, in fact, degenerating metaphases. Compared to Ara-C fewer dead cells were seen in the crypts with the other treatment regimes, though in general the patterns of change were broadly similar.

The light microscopic observations suggested that all drug-induced cell death was occurring through the process of apoptosis, but ultrastructural analysis was necessary to provide unequivocal corroborative evidence to support this hypothesis. Transmission electron microscopic analysis revealed evidence of cell damage as early as 0.5 h following exposure to Ara-C. Even over a 25-fold dose range of Ara-C, all cell death appeared to be achieved by apoptosis (Figures 4 and 5). A well defined halo was present around many dead cell fragments (Figure 4), and condensation and margination of chromatin and the formation of chromatin crescents was frequently seen (Figures 4 and 5). Similar apoptotic profiles were seen after $HN_2$ (Figure 6), ADR (Figure 7) and VCR (Figure 8). At no time did we observe phenomena suggestive of necrosis, such as whole cells with swollen or disrupted organelles or extracellular debris. Overall, EM analysis of all drug-induced damage indicated unequivocally that apoptosis was the sole mode of cell death in this study.

Scanning electron microscopic analysis provided further morphological insights into the apoptotic process. In sectioned crypts many of the apoptotic bodies were seen 'shelled out' from the bounding membrane of the heterophagocytic vacuole (Figure 9). The belief that these spherical bodies were in fact apoptotic bodies was supported by the fact that they were not present in control animals, they only occurred in the locations where, from light microscope observation, damage was expected (cf Figure 2), and finally they had clearly been embedded in enterocyte cytoplasm (in heterophagic vacuoles) as indicated by the 'cups' that cradled them. In other examples (Figure 10), a space was clearly visible between the apoptotic body and the bounding membrane, perhaps explaining the halo commonly seen around phagocytosed apoptotic bodies in LM and TEM preparations.

### Discussion

All the four drugs studied caused cell death in the crypts and this appeared to be achieved preferentially through the process of apoptosis rather than necrosis. Cell death through the apoptotic process is considered as a physiological or near physiological response controlled by extrinsic and ultimately intrinsic mechanisms (Wyllie, 1981; Kerr *et al.*, 1987), yet in the present study apoptosis brought about the massive destruction of cells in a renewing system with potentially lethal implications for the host animal. As such, apoptosis does not seem to be solely a benevolent, physiological response instituted for the removal of unwanted cells in the body. It was also noteworthy that apoptosis was the end-result of toxicity caused by four cytotoxic drugs with completely different modes of action.

Activation of a non lysosomal endonuclease enzyme is considered as the key event in precipitating apoptosis (Arends *et al.*, 1990), and recent observations indicate that many genes may play a part in regulating the process, some of which have a 'protective' effect in preventing premature apoptosis (Buttyan *et al.*, 1988; Buttyan *et al.*, 1989; Debatin *et al.*, 1990; Hockenbery *et al.*, 1990; Williams *et al.*, 1990; Gregory *et al.*, 1991; Williams, 1991; Yonish-Rouach *et al.*, 1991). In some cases lack of specific growth factors has been deemed responsible for activating apoptosis (Williams *et al.*, 1990), whilst in other cases an inappropriate mixture of

**Figure 4** Typical apoptotic body with chromatin margination (C), seen in an enterocyte at 2 h after injection of Ara-C at 40 mg $kg^{-1}$. A clear halo (H) can be seen around the apoptotic body (bar = 1.1 μm).

**Figure 5** Apoptotic bodies (A) containing well preserved organelles in a viable enterocyte 2 h after injection of Ara-C at 1,000 mg kg$^{-1}$. Note the severe distortion of the host cell nucleus, (N) (bar = 1.1 $\mu$m).

extrinsic signals ('unbalanced signalling') can set in train an unopposed cascade of intracellular reactions leading to the same result (McConkey *et al.*, 1990). It is hard to see how the cytotoxic drugs used in the present study could interfere directly with either the availability of intestinal growth factors or the signal transduction mechanisms. Since all the drugs disrupt the passage of cells through the cell cycle, it is more likely that the stimulus for a programme of cell death to be instituted comes from a perturbation of the normally integrated series of cell cycle events as a whole i.e. unbalanced growth.

The most intriguing question which arises from studies such as this, is, – how do such a relatively disparate group of noxious stimuli elicit the same highly conserved response, namely apoptosis? As noted by Dive and Hickman (1991), this is at present an intractable problem as we are largely ignorant as to how the cell 'senses' damage and produces the appropriate 'signal'. In the present study the common factor between the cytotoxic agents is that their cellular targets are intimately involved in cell proliferation, and indeed there are many reports of cell cycle-specific drugs causing apoptosis (Philips & Sternberg, 1975; Searle *et al.*, 1975; Kaufmann, 1989; Barry *et al.*, 1990; Eastman, 1990). On the other hand, even distinctly different cell perturbations like hyperthermia

**Figure 6** Large apoptotic body with numerous intact mitochondria and chromatin fragments (C) in an enterocyte at 2 h after injection of $HN_2$ at $10\,mg\,kg^{-1}$ (bar = 0.8 $\mu$m).

**Figure 7** A single apoptotic body (A), found at the base of the crypt (note Paneth cell granules – P), adjacent to an endocrine cell (E) at 4 h after injection of ADR at $20\,mg\,kg^{-1}$ (bar = 1.8 $\mu$m).

**Figure 8** Numerous apoptotic bodies (*) and arrested metaphases (M), found in enterocytes at 4 h after injection of VCR at 0.4 mg $kg^{-1}$. The fact that very few apoptotic bodies were found until after 2–4 h of metaphase arrest suggests that cells died from metaphase, and not from other phases of the cell cycle (bar = 2.1 μm).

**Figure 9** Scanning electron micrograph of an axially fractured crypt showing the lumen (L) with apoptotic bodies (*), embedded in the cytoplasm of enterocytes. Paneth cell granules (P) are clearly visible at the base of the crypt. From a mouse killed 4 h after injection of Ara-C at 1,000 mg $kg^{-1}$ (bar = 5 μm).

(Takano *et al.*, 1991) and hydrogen peroxide or ethanol (Lennon *et al.*, 1990) can still induce apoptosis.

The major aim of the present experiments was to observe if apoptosis gave way to necrosis as the dose of cytotoxic drug was increased to life-threatening proportions. The amounts administered in the present experiments were much higher than those deemed necessary to cause drug induced apoptosis in the previous studies, and where comparably high doses were used (Ijiri & Potten, 1987), these authors could not unequivocally discriminate between necrosis and apoptosis since only light microscopy was used. Using cell lines from haematological malignancies as the target cells, Lennon *et al.* (1990) did in fact note a definite switch from apoptosis to necrosis as the level of cytotoxic drug was increased. However, we observed no such change in the mode of cell death in intestinal crypt cells with varying dose. Using 10-fold variations in ADR, VCR and $HN_2$, no differences in the mode of cell death were found, and likewise after Ara-C no switch to necrosis was seen with even a 25-fold increase (Figures 4 and 5). These results suggest that therapeutic prevention of drug-induced intestinal toxicity very much depends on understanding the mechanisms which trigger apoptosis.

Thanks to the Association of Commonwealth Universities for granting an ODASSS Award to Dr Anilkumar to undertake his studies in Experimental Pathology at the RPMS.

**Figure 10** Scanning electron micrograph of two transversely sectioned crypts from the same source as Figure 9 showing spherical apoptotic bodies (*), in fractured heterophagic vacuoles. The lumen (L) of each crypt is just visible (bar = 5 μm).

### References

ALISON, M.R. & SARRAF, C.E. (1992). Apoptosis – a type of programmed cell death. *J. Roy. Coll. Phys.* (in press).

ARENDS, M.J., MORRIS, R.G. & WYLLIE, A.H. (1990). Apoptosis: the role of the endonuclease. *Am. J. Pathol.*, **136**, 593.

BENTON, H.P. & ALISON, M.R. (1984). Do cells of continually renewing populations and those stimulated from quiescence respond similarly to HU and Ara-C? *Cancer Chemother. Pharmacol.*, **12**, 53.

BARRY, M.A., BEHNKE, C.A. & EASTMAN, A. (1990). Activation of programmed cell death (apoptosis) by cisplatin, other anticancer drugs, toxins and hyperthermia. *Biochem. Pharmacol.*, **40**, 2353.

BUTTYAN, R., ZAKERI, Z., LOCKSHIN, R. & WOLGEMUTH, D. (1988). Cascade induction of c-*fos*, c-*myc* and heat shock 70K transcripts during regression of rat ventral prostate gland. *Mol. Endocrinol.*, **2**, 650.

BUTTYAN, R., OLSSON, C.A., PINTAR, J. & 4 others (1989). Induction of TRPM-2 gene in cells undergoing programmed death. *Mol. Cell. Biol.*, **9**, 3473.

DEBATIN, K.M., GOLDMANN, C.K., BAMFORD, R., WALDMANN, T.A. & KRAMMER, P.H. (1990). Monoclonal antibody mediated apoptosis in adult T-cell leukaemia. *Lancet*, **335**, 497.

DIVE, C. & HICKMAN, J.A. (1991). Drug-target interactions: only the first step in the commitment to a programmed cell death. *Br. J. Cancer*, **64**, 192.

EASTMAN, A. (1990). Activation of programmed cell death by anticancer agents: cisplatin as a model system. *Cancer Cells*, **2**, 275.

GREGORY, C.D., DIVE, C., HENDERSON, S. & 4 others (1991). Activation of Epstein-Barr virus latent genes protects human B cells from death by apoptosis. *Nature*, **349**, 612.

HOCKENBERY, D., NUNEZ, G., MILLIMAN, C., SCHREIBER, R.D. & KORSMEYER, S.J. (1990). Bcl-2 is an inner mitochondrial membrane protein that blocks programmed cell death. *Nature*, **348**, 334.

IJIRI, K. & POTTEN, C.S. (1983). Response of intestinal cells of differing topographical and hierarchical status to ten cytotoxic drugs and five sources of radiation. *Br. J. Cancer*, **47**, 175.

IJIRI, K. & POTTEN, C.S. (1987). Further studies on the response of intestinal crypt cells of different hierarchical status to eighteen different cytotoxic agents. *Br. J. Cancer*, **55**, 113.

KAUFMANN, S.H. (1989). Induction of endonucleolytic DNA cleavage in human acute myelogenous leukaemia cells by etoposide, camptothecin and other cytotoxic anticancer drugs: a cautionary note. *Cancer Res.*, **49**, 5870.

KERR, J.F.R., WYLLIE, A.H. & CURRIE, A.R. (1972). Apoptosis: a basic biochemical phenomenon with wide-ranging implications in tissue kinetics. *Br. J. Cancer*, **26**, 239.

KERR, J.F.R., SEARLE, J., HARMON, B.V. & BISHOP, C.J. (1987). Apoptosis. In *Perspectives in Mammalian Cell Death*. (ed.) C.S. Potten, pp. 93–128. Oxford University Press, Oxford.

LENNON, S.V., MARTIN, S.J. & COTTER, T.G. (1990). Induction of apoptosis (programmed cell death) in tumour cell lines by widely diverging stimuli. *Biochem. Soc. Trans.*, **18**, 343.

McCONKEY, D.J., ORRENIUS, S. & JONDAL, M. (1990). Cellular signalling in programmed cell death (apoptosis). *Immunol. Today*, **11**, 120.

PHILIPS, F.S. & STERNBERG, S.S. (1975). The lethal actions of antitumor agents in proliferating cell systems *in vivo*. *Am. J. Pathol.*, **81**, 205.

SEARLE, J., LAWSON, T.A., ABBOTT, P.J., HARMON, B. & KERR, J.F.R. (1975). An electron microscope study of the mode of cell death induced by cancer chemotherapeutic agents in populations of proliferating normal and neoplastic cells. *J. Pathol.*, **116**, 129.

TAKANO, Y.S., HARMON, B.V. & KERR, J.F.R. (1991). Apoptosis induced by mild hyperthermia in human and murine tumour cell lines: a study using electron microscopy and DNA gel electrophoresis. *J. Pathol.*, **163**, 329.

WILLIAMS, G.T. (1991). Programmed cell death: apoptosis and oncogenesis. *Cell*, **65**, 1097.

WILLIAMS, G.T., SMITH, C.A., SPOONCER, E., DEXTER, T.M. & TAYLOR, D.R. (1990). Haemopoietic colony stimulating factors promote cell survival by suppressing apoptosis. *Nature*, **343**, 76.

WRIGHT, N.A. & ALISON, M.R. (1984). Kinetic parameters in the gastrointestinal mucosa. In *The Biology of Epithelial Cell Populations*, vol. 2. Clarendon Press, Oxford. pp. 634–687.

WRIGHT, N.A. & AL-NAFUSSI, A. (1982). II. Studies on growth control after death of proliferative cells induced by cytosine arabinoside, with special reference to negative feedback mechanisms. *Cell Tissue Kinet.*, **15**, 611.

WYLLIE, A.H. (1981). Cell death: a new classification separating apoptosis from necrosis. In *Cell Death in Biology and Pathology*. Bowen, I.D. & Lockshin, R.A. (eds). pp. 9–34. Chapman and Hall: London.

WYLLIE, A.H., KERR, J.F.R. & CURRIE, A.R. (1980). Cell death: the significance of apoptosis. *Int. Rev. Cytol.*, **68**, 251.

YONISH-ROUACH, E., RESNITZKY, D., LOTEM, J., SACHS, L., KIMCHI, A. & OREN, M. (1991). Wild type p53 induces apoptosis of myeloid leukaemic cells that is inhibited by interleukin-6. *Nature*, **352**, 345.